# Can ECIS Biosensor Technology Be Used to Measure the Cellular Responses of Glioblastoma Stem Cells?

**DOI:** 10.3390/bios11120498

**Published:** 2021-12-06

**Authors:** Laverne Diana Robilliard, Jane Yu, Sung-Min Jun, Akshata Anchan, Graeme Finlay, Catherine E. Angel, Euan Scott Graham

**Affiliations:** 1 Department of Molecular Medicine and Pathology, School of Medical Sciences, Faculty of Medical and Health Sciences, University of Auckland, Auckland 1025, North Island, New Zealand; l.robilliard@auckland.ac.nz (L.D.R.); jane.yu@auckland.ac.nz (J.Y.); sjun881@aucklanduni.ac.nz (S.-M.J.); a.anchan@auckland.ac.nz (A.A.); g.finlay@auckland.ac.nz (G.F.); 2 Centre for Brain Research, University of Auckland, Auckland 1025, North Island, New Zealand; 3 Auckland Cancer Society Research Centre, Faculty of Medical and Health Sciences, University of Auckland, Auckland 1025, North Island, New Zealand; 4 School of Biological Sciences, Faculty of Science, University of Auckland, Auckland 1025, North Island, New Zealand; c.angel@auckland.ac.nz

**Keywords:** ECIS technology, impedance, glioblastoma, adhesion, migration, wound healing

## Abstract

Glioblastoma is considered the most aggressive and lethal form of brain cancer. Glioblastoma tumours are complex, comprising a spectrum of oncogenically transformed cells displaying distinct phenotypes. These can be generated in culture and are called differentiated-glioblastoma cells and glioblastoma stem cells. These cells are phenotypically and functionally distinct, where the stem-like glioblastoma cells give rise to and perpetuate the tumour. Electric cell-substrate impedance sensing (ECIS) is a real-time, label-free, impedance-based method for the analysis of cellular behaviour, based on cellular adhesion. Therefore, we asked the question of whether ECIS was suitable for, and capable of measuring the adhesion of glioblastoma cells. The goal was to identify whether ECIS was capable of measuring glioblastoma cell adhesion, with a particular focus on the glioblastoma stem cells. We reveal that ECIS reliably measures adhesion of the differentiated glioblastoma cells on various array types. We also demonstrate the ability of ECIS to measure the migratory behaviour of differentiated glioblastoma cells onto ECIS electrodes post-ablation. Although the glioblastoma stem cells are adherent, ECIS is substantially less capable at reliably measuring their adhesion, compared with the differentiated counterparts. This means that ECIS has applicability for some glioblastoma cultures but much less utility for weakly adherent stem cell counterparts.

## 1. Introduction

Glioblastoma is one of the most devastating forms of brain cancer, owing to the lack of treatment options, complexity of the tumour locations restricting surgical options, and the dismal survival statistics [1]. Glioblastoma tumours are highly heterogeneous [2,3], and comprise transformed cells driven by oncogenic mutations, which exist in a range of different phenotypic and functional forms [4]. In addition to these cancer cells, this brain tumour also contains a variety of cells including myeloid derived suppressor cells, regulatory T cells, and aberrant vascular beds [2,4]. The devastating nature of this tumour is exemplified by the fact that complete surgical removal of the tumour is never 100% achievable [5] as it always relapses. This implies that there are niches of tumour stem-like cells that exist beyond the surgical boundaries [4,6]. These potentially reseed the tumour with devastating effects. This research relates to our strategy of measuring killing of glioblastoma cells and measuring their invasive or migratory potential. ECIS technology offers an impedance-based solution with real-time autonomous capability [7,8].

**Figure 1:**
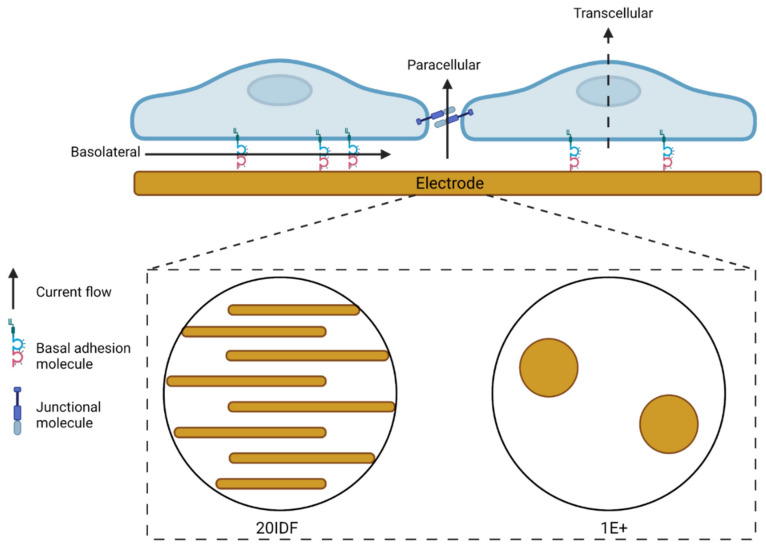
Electric cell-substrate impedance sensing (ECIS) schematic of current flow. Resistance measurements acquired by ECIS are a consequence of current flow through multiple pathways. Current flows through the basolateral, paracellular, and transcellular compartments. The cellular biology dictates the pathway that provides the most resistance to current flow. Current flow is impeded by the presence of strong basal adhesion proteins, paracellular junctional proteins and the cell body atop the electrode. ECIS electrode configurations include, but are not limited to, an interdigitated orientation of electrodes that measure a large surface area of a well, or two small circular electrodes that measure a comparatively smaller surface area of a well. Consult the Applied Biophysics website (https://www.biophysics.com/; accessed on 19 October 2021) for the most up-to-date array types and configurations.

ECIS uses real-time, impedance-based recordings of cell adhesion, growth and motility over gold electrodes embedded into the bottom of a custom-made 96- or 8-well plates [9]. The sensitivity of the biosensor technology heavily relies on both the electrode and cell properties. Cells seeded onto the custom plates settle and adhere to the electrode arrays. A weak alternating current is passed through the electrode arrays, either at a single frequency or multi-frequencies (250 Hz–64,000 Hz). The cells, depending on their properties, impede the free flow of current through the electrodes (see Figure 1). This impedance is measured, and the resistive properties of the cells can be used to infer changes to various cell properties over time. Cells with high adhesive properties, for example, will impede the flow of current to a greater extent than cells with low adhesive properties [9]. Depending on the electrode arrays used, different numbers of cells are measured. Using a 96W20IDF interdigitated array, a large surface area (2.09 mm^2^) of the well is covered, thus many cells are recorded (2000–4000). Alternatively, using a 96W1E+ array, only 0.256 mm^2^ (2 circular electrodes) are of the well is covered by electrode, measuring ~100–200 cells. Comparatively, 8W1E arrays records 50–100 cells over a 0.049 mm^2^ electrode area.

**Figure 2:**
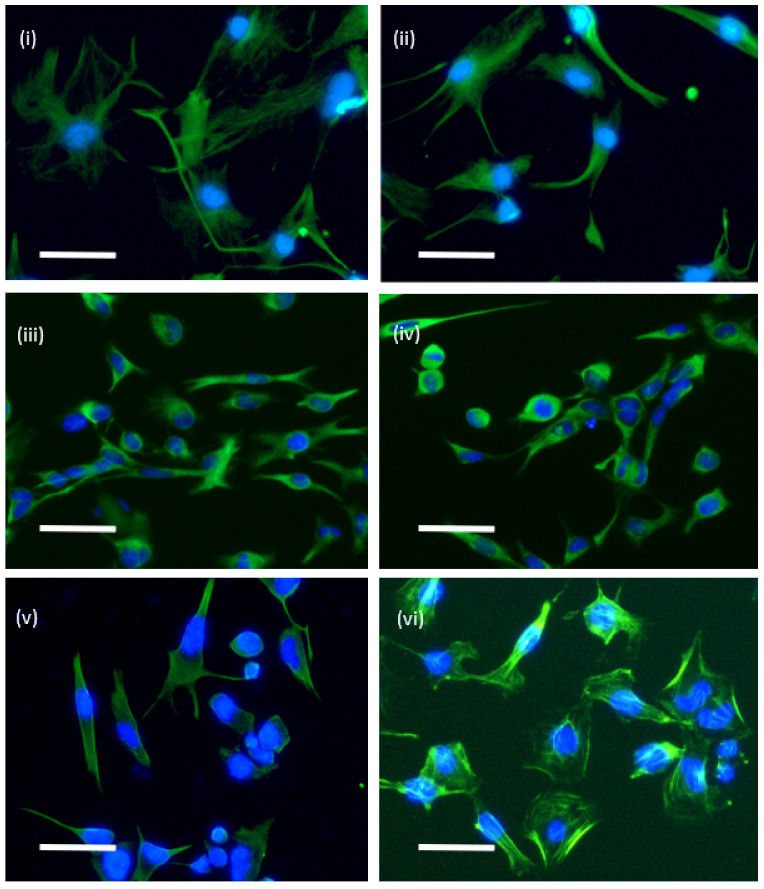
Morphology of adherent glioblastoma stem-like cells. The cells were stained for various cytoskeletal intermediate filaments to optimally reveal the cellular morphology and adherent shape of the cells. Cells were seeded at low density (5000 cells per well) and grown on laminin for 2–3 days. All images are from stem cell cultures of (**i**) and (**ii**) NZB11 stained for nestin, (**iii**) and (**iv**) NZB19 stained for nestin, (**v**) NZB12 stained for nestin, and (**vi**) NZB12 stained for actin. Scale bar is 50 µm.

We have previously used ECIS technology, with considerable success, to measure the adhesion and barrier integrity of brain endothelial cells [9,10,11,12,13]. We postulated from this prior work that ECIS technology could offer the perfect solution for (i) measuring killing of glioblastoma cells, and (ii) also for monitoring the migratory kinetics of the glioblastoma cells. In the context of glioblastoma cells, they exist as a spectrum of phenotypes within the tumour and can be recapitulated in vitro as (i) serum-differentiated cells and (ii) stem-like cells [14]. Our previous work has shown that the serum-differentiated cells and stem-like glioblastoma cells grow well as adherent cells [14]. We have noted previously that the stem-like cells are typically smaller than their serum-differentiated counterparts. Figure 2 exemplifies the adherent but highly variable morphology of the adherent glioblastoma stem-like cells. Based on the adherent nature of these cells, we hypothesised that ECIS biosensor technology would be sensitive enough to measure the adherent characteristics of both types of cultures. We were particularly excited about the potential of ECIS for measuring the killing of the stem-like glioblastoma cells, which is an area of considerable interest therapeutically. Targeting the stem cell niche is particularly relevant for precision killing (e.g., NK-mediated or cytotoxic drugs) and preventing migration of these cells into new healthy brain regions. In this context, the adherent properties of the cells are used as a surrogate of compromise and killing, and biosensor technology provides a valuable real-time assessment of when this occurs.

In this study, we reveal that ECIS has considerable potential to measure the adherent characteristics of the serum-differentiated phenotypes of glioblastoma cells. We also reveal the potential of ECIS to monitor the migratory behaviour of these cells. This could be applied to a range of drug discovery approaches in the future. However, we were unable to reliably detect a strong or stable level of adhesion from the stem-like glioblastoma cells. This was an unexpected observation, as the stem-like cells do adhere. This therefore limits the utility of ECIS for future investigations (e.g., killing assays) using the stem-like glioblastoma cells. However, it does highlight the important diversity in phenotype, where although the stem cell counterparts are adherent, the nature of the adhesion is considerably different to the serum-differentiated cells.

## 2. Materials and Methods

### 2.1. Cell Culture

Early Passage New Zealand Glioblastoma cell lines. NZB11, NZB12, NZB13, and NZB19 cell lines were provided in collaboration with the Auckland Cancer Society Research Centre. The cells were cultured at 37 °C in 5% O_2_, 5% CO_2_ as adherent monolayers on uncoated 25 cm^2^ culture flasks until 80–90% confluent in alpha-Minimal Essential Medium (MEM) (ThermoFisher, Auckland New Zealand) supplemented with serum (5% FBS (Moregate)) and 1× insulin-transferrin-selenium (ITS) (Sigma) (herein referred to as serum-derived cultures).

Adherent GBM Cancer Stem Cell-Like Cells (*gCSC*). Adherent gCSCs were expanded for experimental use and routinely cultured at 37 °C in 5% O_2_, 5% CO_2_. NZB11, NZB19, NZB12, and NZB13 cell lines were cultured in 25 cm^2^ culture flasks coated with 10 μg/mL laminin (ThermoFisher, Auckland, New Zealand) in Dulbecco’s Modified Eagle Medium/F12 (DMEM/F12) (ThermoFisher, Auckland, New Zealand) supplemented with 0.5× B-27 minus vitamin A (ThermoFisher, Auckland New Zealand), 0.5× N_2_ supplement (ThermoFisher, Auckland New Zealand), 20 ng/mL bFGF (Peprotech), and 20 ng/mL EGF (Novus Biologicals) (herein referred to as gCSC cultures). The phenotypes of the serum-derived and gCSC cultures have been detailed extensively [14].

### 2.2. Immunocytochemistry of Cytoskeletal Proteins

For immunocytochemistry, glioblastoma stem-like cells were seeded at low density of 5000 cells per well (96-well plate). Cells were grown for 2–3 days on laminin and then fixed with 4% paraformaldehyde (PFA) for 10 min at room temperature. PFA was washed off with 1× PBS and then cells were permeabilised with PBS-0.1% Triton X-100 (PBS-T). Cells were stained for cytoskeletal structures to reveal cellular morphology. Cells were incubated with anti-nestin (sc-23927) at 1:100 titration for 1 h at room temperature with gentle agitation. After which, non-bound antibody was removed, cells were washed thrice with PBS-T, and then incubated with AlexaFlour488 conjugated secondary (anti-mouse) at 1:400 titration for 1 h at room temperature. Alternatively, cells were stained with ActinGreen^TM^ 488 ReadyProbes^TM^, as per the manufacturer’s protocol. Cells were counterstained with 1:10,000 Hoechst 33342 (ThermoFisher). For imaging, an EVOS FL-auto imaging system (ThermoFisher) was used.

### 2.3. Electric Cell-Substrate Impedance Sensing (ECIS)

ECIS technology was used to measure glioblastoma serum-derived and gCSC adhesion profiles and migratory dynamics. Glioblastoma serum-derived and gCSC cells were harvested by adding Accutase (ThermoFisher, Auckland, New Zealand) for 2 min. Accutase was diluted in 1:1 cell culture medium and cells were pelleted at 300× *g* for 5 min. The cell pellet was resuspended in 1 mL of medium and viable cells were counted using trypan blue exclusion (ThermoFisher, Auckland New Zealand). Cells were then seeded onto either 96W20IDF PET (Applied BioPhysics, New York, NY, USA), 96W1E+ PET (Applied BioPhysics, New York, NY, USA), or 8W1E PET (Applied biophysics, New York, NY, USA) arrays at 80,000 cells per 0.33 cm^2^. Images of the electrode architecture can be found on the Applied Biophysics website (see https://www.biophysics.com/cultureware.php; accessed on 19 October 2021). gCSC cells were seeded onto 10 µg/mL laminin to promote adhesion. Glioblastoma cells were grown in culture medium for 60–140 h (until a maximum resistance, Ω, was recorded). For wounding on both 96W1E+ and 8W1E arrays, selected wells were wounded by passing a 100 kHz current at 1800 µA for 120 s. Impedance measurements were extracted from Applied BioPhysics ECIS software v1.2.186.0 PC and plots were created in GraphPad Prism v.7. Live phase contrast images were acquired immediately following the end of ECIS recording periods using an Olympus CKX53 light microscope.

Following completion of ECIS experiments, cells were then fixed in 4% paraformaldehyde (PFA) for 10 min and then washed once with PBS. Cells fixed in PFA were permeabilised for a further 10 min in PBS-0.1% Triton X-100 (PBS-T). Cells were washed and stored in PBS. For immunocytochemical analysis, PBS was aspirated, and cells were blocked in 1% Bovine Serum Albumin (BSA) for 45 min and washed thrice for 10 min in 0.1% PBS-T. Cells were stained with ActinGreen^TM^ 488 ReadyProbes^TM^, as per the manufacturer’s protocol. Cells were counterstained with 1:10,000 Hoechst 33342 (ThermoFisher). Cells were then washed as previously described and stored in PBS. For imaging, an EVOS FL auto imaging system (ThermoFisher) was used. Images were acquired using GFP, DAPI, and phase filters at 20× magnification.

### 2.4. NanoString Gene Expression Analysis of Glioblastoma Expressed Adhesion Molecules

NanoString^TM^ gene expression analysis used a custom designed codeset panel containing probes for glioblastoma cadherins, integrins and 4 housekeeping genes (see Table 1). RNA from cell pellets was isolated using RNAqueous^TM^—Micro Total RNA Isolation Kit (cat. No. AM1931, ThermoFisher). RNA quality and quantity were determined using NanoDrop^TM^ and Agilent RNA Screentape^®^. 200 ng of RNA was analysed using the nCounter platform and output data was analysed using nSolver 4.0 advanced analysis. NanoStringTM analysis was conducted by Grafton Clinical Genomics (https://www.graftonclinicalgenomics.ac.nz/; accessed on 19 October 2021) at the University of Auckland. Data are presented as mRNA counts.

## 3. Results

### 3.1. Serum-Derived Glioblastoma Cell Adhesion Can Be Monitored Using 20IDF ECIS Technology

**Figure 3:**
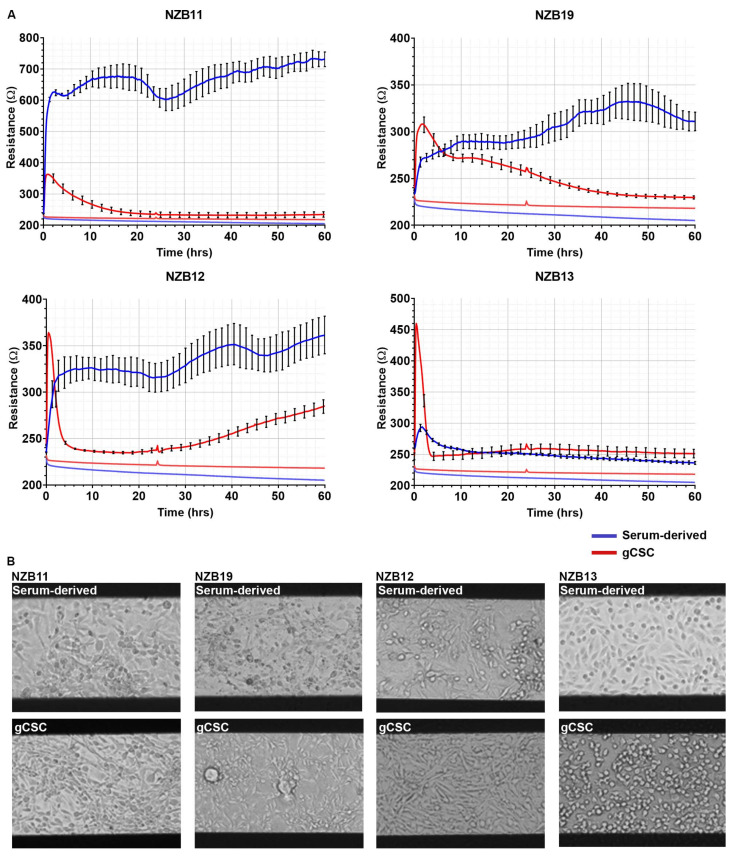
Serum-derived and gCSC cell resistance on 96W20IDF ECIS arrays. (**A**) Resistance measurements at 4000 Hz over 60 h of growth. Comparison of adhesion profiles of NZB11, NZB12, NZB19, and NZB13 serum-derived and gCSC cells seeded at 80,000 cells. Adhesion profiles referenced against a cell-free control (bottom flat red and blue lines) are shown. Data are representative of three independent replicates. (**B**) Phase contrast images of NZB11, NZB12, NZB19, and NZB13 serum-differentiated and gCSC cells after 60 h of growth on 96W20IDF ECIS arrays. The dark bands are the interdigitated electrodes. Images acquired at 20× magnification. Data are representative of three independent replicates.

The primary assessment was to determine whether ECIS technology could robustly monitor the adhesion characteristics (see Figure 1) of glioblastoma cells using standard 96-well 20IDF arrays. Each well has an electrode area of 3.985 mm^2^ comprised of interdigitating electrodes. Figure 3 reveals the adhesion profiles of 4 different patient-derived glioblastoma lines, for both serum-differentiated cells and the glioblastoma stem cell counterparts. For each culture, adhesion of the cells to the arrays is fast and can be detected within the first hour of seeding the cells. This implies that the adhesion molecules used by the glioblastoma cells are constitutively expressed and unaffected by the harvesting of the cells.

The major observation from these data is that adhesion of the NZB11, NZB12, and NZB19 serum-differentiated cells is considerably stronger and more stable than the stem cell equivalents. Notably NZB11, NZB12, NZB13, and NZB19 stem cell cultures all, initially, have strong basolateral adhesion; however, this measurement is transient and drops off rapidly for NZB12 and NZB13 (Figure 3). The adhesion level drops off for NZB11 and NZB19 stem cell cultures to levels indistinguishable to the cell-free well. This potentially relates to the formation of glioblastoma colonies (only occurs in the stem cell cultures) and thus, depletion of cells from the electrodes. The phase images of the glioblastoma cultures clearly demonstrate that the glioblastoma cells are adherent; however, ECIS demonstrates that the adhesion of the serum-differentiated cells is considerably stronger than the stem cell-like glioblastoma cells. These data highlight the variation between cultures and the limited options for using ECIS to measure cellular responses from the glioblastoma stem cell cultures.

### 3.2. Serum Differentiated Glioblastoma Cells Produce Stronger and More Stable Adhesion on 1E+ Arrays

**Figure 4:**
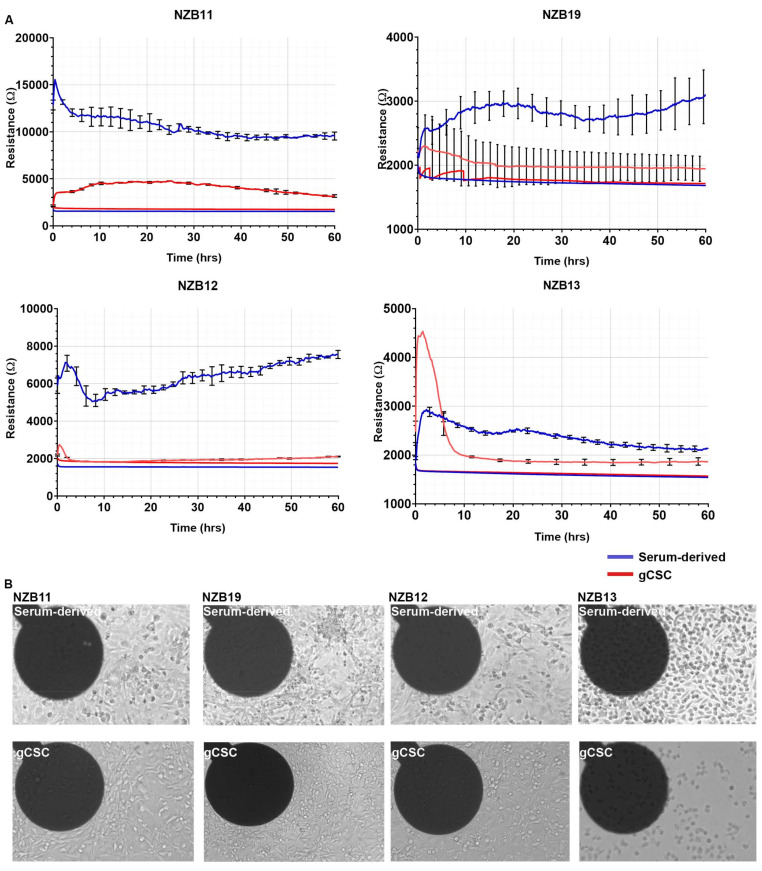
Serum-derived and gCSC cell resistance on 96W1E+ ECIS arrays. (**A**) Resistance measurements at 4000 Hz over 60 h of growth. Comparison of adhesion profiles of NZB11, NZB12, NZB19, and NZB13 serum-derived and gCSC cells seeded at 80,000 cells per well. Adhesion profiles referenced against a cell-free well control (bottom flat red and blue lines) are shown. Data are representative of three independent replicates. (**B**) Phase contrast images of NZB11, NZB12, NZB19, and NZB13 serum-derived and gCSC cells after 60 h of growth on 96W1E+ ECIS arrays. Dark circles are recording electrode regions. Images acquired at 20× magnification. Data is representative of three independent replicates. See Supplementary Figure S1 for a contrast-adjusted zoom of the electrodes, which reveals adherent cells on each.

We next conducted the same analysis using 96-well 1E+ ECIS arrays rather than the 96-well 20IDF arrays. The 1E+ arrays have 2 circular electrodes per well comprising an area of 0.256 mm^2^. The rationale for this was to see if the glioblastoma stem cell adhesion was more stable with a single electrode. If the rapid drop in resistance was due to sphere formation and loss of cells on the larger electrode array (20IDF), a single electrode could potentially capture signal for longer. As with the 20IDF arrays, glioblastoma cells adhesion to the single 1E+ electrode was rapid and strong (initially) for most of the cultures (Figure 4A). It should be highlighted that the cell-free resistance from the 1E+ arrays was ~2000 ohms (Figure 4), much greater than that observed from the 20IDF arrays (Figure 3).

In general terms, the resistance measurements indicate that the adhesion of the serum-differentiated cells was considerably stronger than the stem cell cultures (Figure 4A). NZB11, NZB12, and NZB19 each provided a good temporal window of at least 60 h for experimental stimulation, which is ideal for future cytotoxicity studies (e.g., conducting NK killing effector studies or drug-induced cytotoxicity). For the 1E+ arrays, a good stable signal was produced for the NZB11 stem cell cultures, whereas the signal from the NZB12 and NZB19 was indistinguishable from the cell-free wells. Intriguingly, NZB13 cells have a very high resistance initially, suggesting a very strong level of adhesion to the electrode for the first 2–6 h, after which this signal is lost.

This implies that the molecular phenotype of the NZB13 cells changes dynamically and rapidly, where initially the cells have a high capacity to adhere and then become less adherent. This postulate is intriguing and consistent with the molecular capability of glioblastoma stem cells but is outside the scope of this paper. There was also a lack of observable spheres in the NZB13 cultures during this time frame, excluding the earlier consideration of cell loss into spheres (Figure 4B). The phase contrast images (Figure 4B) clearly reveal the adherent nature of the stem-like cells, and Supplementary Figure S1 (contrast enhanced) reveals the presence of the glioblastoma stem-like cells on the electrodes at the 60-hour time point. So, the lack of impedance measurement is not due to a complete absence of the cells on the electrode surface.

### 3.3. ECIS Reveals Pronounced Variation in Adhesion Properties across Experiments

**Figure 5:**
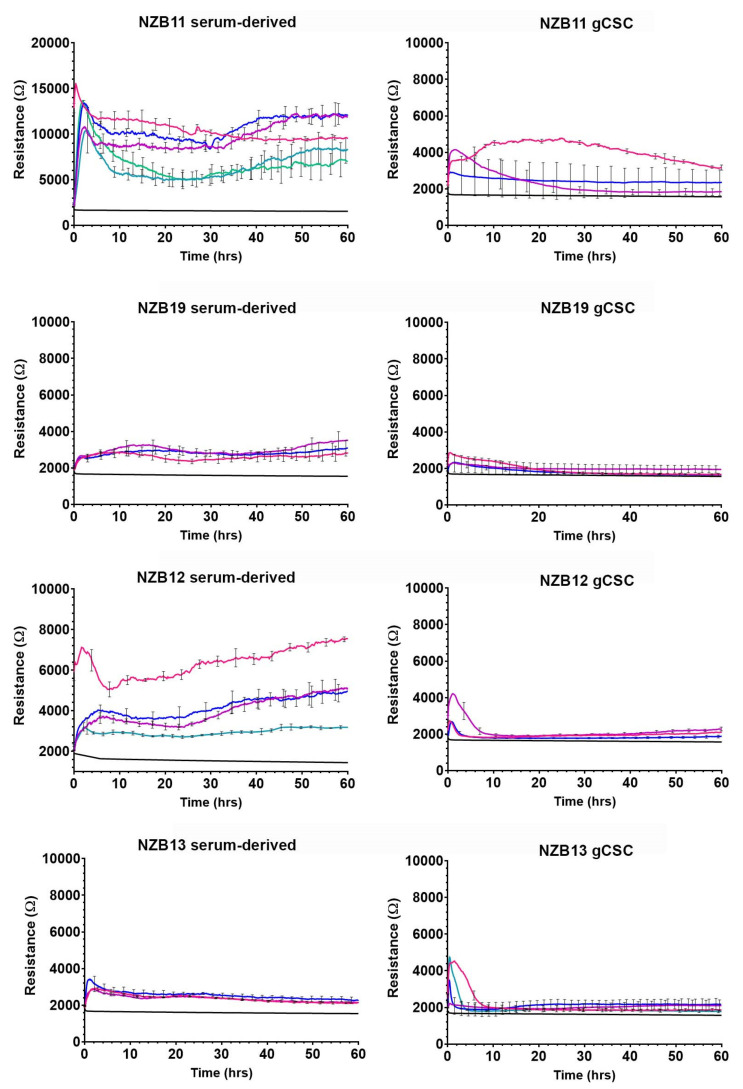
Representation of inter-experimental variability of serum-derived and gCSC cells grown on 96W1E+ ECIS arrays. Resistance measurements at 4000 Hz over 60 h of growth. Comparison of adhesion of NZB11, NZB12, NZB19, and NZB13 serum-derived and gCSC cells seeded at 80,000 cells. Growth profiles, referenced against a cell-free control well (black line), are shown. Each coloured adhesion profile represents an independent replicate.

The data shown in Figure 5 highlights the variation and consistency of resistance indicative of overall cellular adhesion for multiple independent experiments for each culture. For these experiments, 96-well 1E+ arrays were used (same array configuration as in Figure 4). For the NZB11 and the NZB12 serum-derived cells, considerable variation in the strength of adhesion was observed across experiments. In Figure 5, each curve represents an independent experiment. In all experiments, identical numbers of cells were seeded initially so the difference in resistance is unlikely due to cell number, but more likely differences in the phenotype of the glioblastoma cells between experiments. This could suggest that the NZB11 and NZB12 cultures have a more dynamic phenotype, where variation in cell adhesion molecule expression between cultures would explain this variation. It is our preference to show the raw resistance values as it reveals this important experimental variation and potential phenotypic variation. This important information would be lost with ECIS data that has been normalised.

For the NZB11 gCSC cultures, there was only one experiment where a good level of adhesion was detected throughout the experiment. This, however, was not observed in any other independent experiment. For each of the other gCSC cultures, modest adhesion was detected in the first few hours but dissipated quickly. These data reveal the inability of ECIS to measure the basolateral adhesion of the glioblastoma stem cell cultures, thus limiting its application in future NK killing or cytotoxicity assays.

### 3.4. Ablation of Serum-Derived Glioblastoma Adhesion and Repopulation on ECIS Arrays

**Figure 6:**
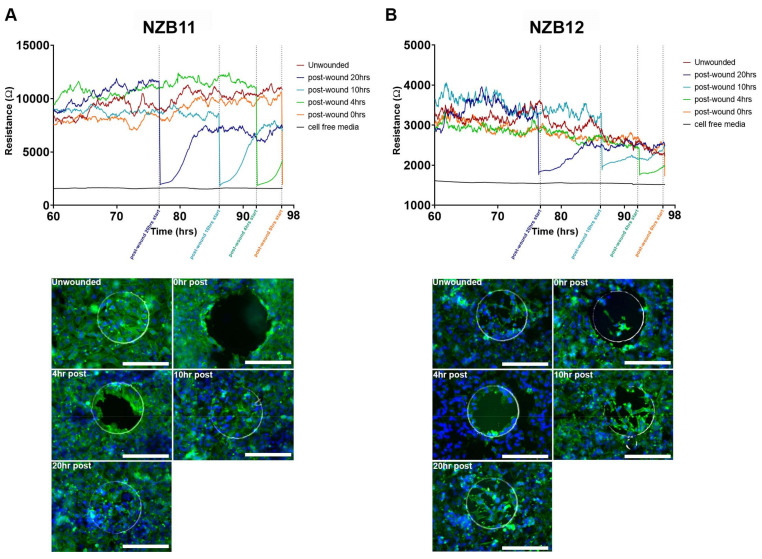
Wound healing profiles of serum-derived GBM cells. (**A**) NZB11 and NZB12 serum-derived cells grown on 8W1E ECIS arrays for 76 h (time point at which stable resistance measurements are acquired). Cells were seeded at 80,000 cells per 0.33 cm^2^. Wounding was induced at 76, 86, 92, and 96 h post-seeding. Wound healing measurements were carried out for 20, 10, 4, and 0 h, corresponding to each wounding time point. Data shows resistance measurements acquired between 60–98 h. Data are representative of three independent replicates. (**B**) Representative fluorescent images show electrode regions 0, 4, 10, and 20 h post-wounding. Unwounded control shown for reference. Cells were stained with Actin Green™ 488 (green) and Hoechst 33342 (blue). Phase electrode ring shows the periphery of each electrode. Images acquired at 20× magnification. Scale bar = 200 µm. Data is representative of three independent replicates.

A unique property of ECIS technology is the capability of delivering a large current to the cells grown on the electrode array. Theoretically, this current should be sufficient to obliterate the cells quickly, resulting in their death and removal from the electrode. Thus, the rationale to this approach is for the study of cell migration kinetics post-ablation. For these experiments, 8-well 1E arrays were used. These have a single circular electrode of 0.049 mm^2^ and typically will measure adhesion from ~50 glioblastoma cells. Figure 6 shows that there is considerable variation across a single array with only eight wells. This is presumably due to the small area of the electrode and consequently the small sampling size. The 1E configuration does not come in a 96-well version, which has implications for design of experiments investigating pharmacologic concentration response profiles, which would require higher throughput (more wells).

In terms of cellular ablation, the electrode impedance is lost instantaneously following application of the current (Figure 6). This implies that the cells are killed instantly and lost from the electrode. The imaging reveals that this is indeed the case, where the electrode is fully covered prior to ablation and then the electrode is clear of nuclei and cellular material immediately after ablation (time 0). Within 4 h there is notable migration of cells back onto the electrode which is then mostly covered within ~10 h. However, although the electrode surface is covered, the strength of the cellular adhesion is not as high post-ablation compared with prior to ablation. The obvious explanation for this is that migratory cells are going to be less adherent than non-migratory cells due to changes in adhesion molecule expression.

A second observation worth highlighting is that the partial repopulation of the electrode is approximately 6–8 h, which is influenced by the area of the electrode. Thus, for the 1E electrode and these cultures of glioblastoma cells there is a relatively short window of investigation for drug-based studies probing migratory kinetics.

**Figure 7:**
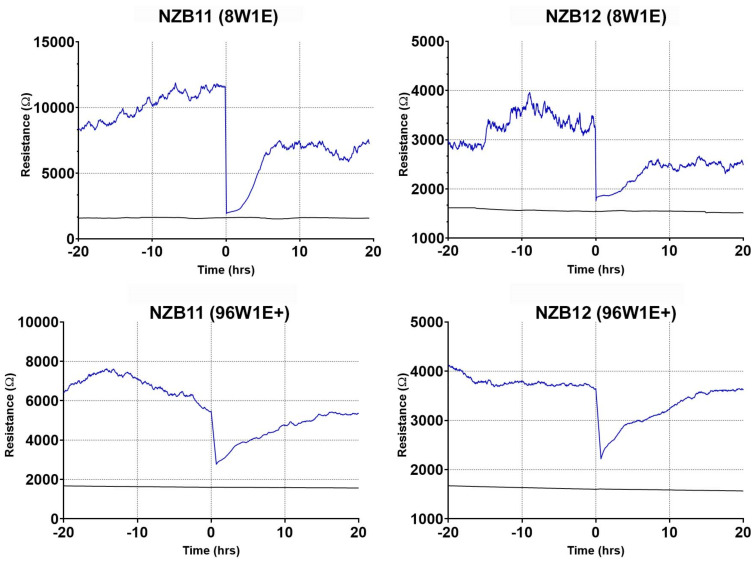
Comparative wound healing profiles of serum-derived GBM cells on 96W1E+ and 8W1E ECIS arrays. NZB11 and NZB12 serum-derived cells grown on 8W1E ECIS arrays for 76 h (time point at which stable resistance measurements are acquired). NZB11 and NZB12 serum-derived cells grown on 96W1E+ ECIS arrays for 95 h (time point at which stable resistance measurements are acquired). Cells were seeded at 80,000 cells per 0.33 cm^2^. Time 0 represent the point that the wounding current was applied. Resistance measurements were acquired at 4000 Hz. Data are representative of three independent replicates.

In Figure 7**,** we reveal the temporal comparison of ablation of the glioblastoma cells on the small 1E electrodes compared with the larger 1E+ electrodes. Both approaches reveal that much of the cellular adhesion is removed immediately after ablation. As expected, it takes considerably longer for the glioblastoma cells to migrate and populate the 1E+ electrode as their area is much larger. This provides multiple options offering different temporal windows, for conducting wound healing or migration-based experiments with highly adherent glioblastoma cells. 

Finally, expression of key integrin and cadherin cell adhesion molecules were measured using NanoString technology. NanoString measures the absolute mRNA count in a sample and is therefore an ideal assessment tool for determining the gene expression profile for a focused range of genes across multiple samples. Here, we were looking for a differential gene expression profile that would explain the weaker extent of adhesion observed in the glioblastoma stem-like cultures.

**Figure 8:**
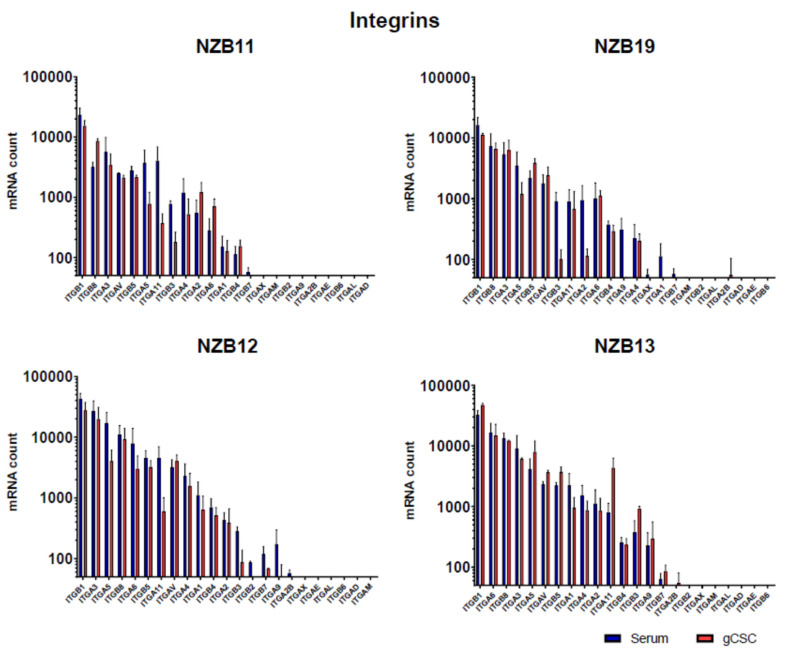
Integrin gene expression by glioblastoma serum-derived and gCSC cells. NanoStringTM analysis of absolute mRNA count in NZB11, NZB19, NZB12, and NZB13 serum-derived and gCSC GBM lines. The results of two independent experiments are shown. Unpaired students t-test analysis was carried out as a comparison of the serum vs gCSC mRNA count where *p*-value ≤ 0.05. None of the comparisons for any of the genes were significant.

NanoString analysis revealed that the glioblastoma cells express a broad range of integrin subunits at very high levels. Notably, the beta-1 subunit was the highest express integrin subunit expressed across all samples. This was followed closely by the beta-8, alpha-3, alpha-5, and the beta-5 subunits. NanoString analysis reveals a range of subunits where expression was not detected at the mRNA level. However, as can be seen in Figure 8 (integrin profile), there was very little difference in mRNA counts between the serum-differentiated cells and the glioblastoma stem-like cells. This was a surprising observation.

**Figure 9:**
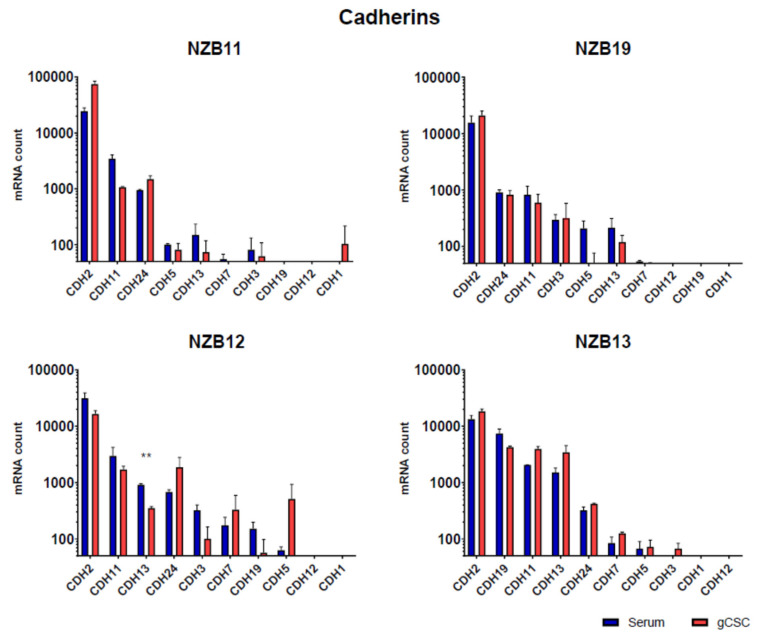
Cadherin gene expression by glioblastoma cells. NanoString^TM^ analysis of absolute mRNA counts in NZB11, NZB19, NZB12, and NZB13 serum-derived and gCSC GBM lines. The results of two independent experiments are shown. Unpaired students t-test analysis was carried out comparing the serum vs gCSC mRNA count for each gene where *p*-value ≤ 0.05 (*) and 0.01 (**).

Cadherin expression (Figure 9) was also analysed, and a similar outcome was observed. Across each of the lines, cadherin 2, 11, 13, and 24 were consistently amongst the top 5 cadherin genes expressed in each line. Whereas cadherin 1 and cadherin 12 were the least abundant at the mRNA level. In terms of differences in mRNA levels between the serum-differentiated and stem-like cultures, the profiles were more similar than different. Overall, this observation was surprising and did not easily explain why the adhesion of the stem-like glioblastoma cells does impede the current flow across the ECIS electrodes and affect the resistance.

## 4. Discussion

The goal of this work was to determine whether ECIS technology could measure the adherent properties of glioblastoma cells. Glioblastoma is the most common and most lethal form of brain cancer [1]. The lethal nature of glioblastoma is highly complex but is a combination of the ability of glioblastoma cells to (i) evade and suppress the host immune system [15,16,17] and the fact that (ii) glioblastoma is a highly invasive cancer [18], which is often not easily demarcated for complete surgical resection. In addition, glioblastoma tumours comprise a heterogeneous mixture of oncogenically transformed cells on a spectrum of phenotypes that have different effector functions [19]. These phenotypes are reflected in the serum-derived cultures and the stem cell-enriched culture. In terms of clinical relevance, there is substantial interest in being able to target and kill the glioblastoma cells that reseed the tumour microenvironment post-surgery or post-therapy. These cells have more of a stem cell phenotype and can generate 3D tumour spheres in vitro and tumours in vivo in mice [20,21].

We are particularly interested in developing strategies to kill glioblastoma cells, particularly the stem cell counterparts and ECIS technology is a potential surrogate for measuring cell death as a function of adhesion. Herein we demonstrate that ECIS technology, using multiple different array configurations, is well suited to measuring and monitoring the long-term adhesive properties of serum-derived glioblastoma cells. These cells are generally highly adherent and provide strong adhesion to the 96-well 20IFD arrays, 96-well 1E+ arrays, and the 8-well 1E arrays. Of note was the ability of the 1E+ arrays to detect inter-experimental variation across independent experiments. This was particularly notable for the NZB11 and the NZB12 serum-derived cells. Although a strong level of adhesion was observed in all experiments, it varied by 5000–6000 ohms for the NZB11 cells and ~3000–4000 ohms for the NZB12 cultures. The advantage of the 96-well array format is that is affords a higher degree of experimental replicates on the plate and this is highly recommended for phenotypically dynamic cells such as these.

In contrast, we were unable to consistently detect a strong stable level of adhesion from the glioblastoma stem-like cultures on any of the different array modalities. This was conducted with seeding numbers that equate to complete coverage of the area of the well, which had been previously optimised [14]. The inability to detect gCSC adhesion using ECIS was most unexpected because we knew that the glioblastoma stem cultures grew as adherent cells on laminin. Imaging of the glioblastoma cells revealed their presence on the electrodes, and we had previously observed firmly adhered cells post-staining protocols, which implied a reasonable level of adhesion. So not only was this an unexpected finding, it was also disappointing, as it precludes the use of ECIS in studying the biology of the cells based on their adherent properties.

The question remains, however, why the stem-like cells did not affect the electrical current across the ECIS electrodes, even though there was adhesion. Analysis of gene expression for key adhesion molecules was conducted using the NanoString platform. Essentially, NanoString technology directly counts the abundance of specific mRNA molecules using custom probe sets. Here, we measured expression of 23 integrin subunits and 10 cadherin genes. These represent a broad spectrum of key adhesion molecules known to interact with ligands present in the extracellular matrix (e.g., laminin, fibrinogen, and collagen). This analysis revealed in interesting profile of both integrin and cadherin expression. It is worth noting the high level of beta-1 integrin subunit expression, the key beta subunit in the VLA (very late antigen) family members, which interact with numerous ligands including laminins, collagens, and fibronectin. However, NanoString analysis did not reveal a significant difference at the mRNA level that would explain the differential adhesion strength measured by ECIS.

This suggests that, although the glioblastoma gCSCs are able to adhere weakly, the molecular repertoire they use must be different to the more adherent serum-derived phenotypes. However, not in a manner that is simply explained at the mRNA level. For our studies, this therefore means that ECIS technology has limited applicability for measuring killing or migration of the glioblastoma stem-like cells. For any given cancer-cell culture or any other cell line this will need to be empirically determined and optimised.

As mentioned previously, the migration and invasiveness of glioblastoma cells are important aspects of their biology. We postulated that the ablation function of ECIS could be used to completely remove cell coverage from the electrode and then monitor migration of surrounding glioblastoma cells into the ablated region. This could be considered analogous to acute surgical resection in a dish. Initially, we assessed this on the 8-well 1E arrays which have a single electrode with a relatively small area, which we thought would maximise cell ablation. This was indeed the case, where complete removal of the cells was observed instantaneously. It was particularly surprising just how fast the glioblastoma cells were able to spread from the non-ablated zone, then migrate and repopulate the electrode. For NZB11, effectively covering the electrode within 10 h. Whereas the migration of NZB12 was quite different and complete repopulation of the electrode was not achieved. It is worth noting that the cellular morphology and actin organisation in the non-ablated cells are markedly different to that of the migrating cells. The glioblastoma cells that migrated onto the electrodes have a more compact cell-body and disorganised actin arrangement. This potentially explains why the level of adhesion post-migration is considerably lower than at the point of ablation. In addition, it is expected that the adhesion profile of migratory cells will be less than that of static or firmly attached non-moving cells. It was also possible to monitor the migratory kinetics of the glioblastoma cells using the larger 1E+ electrode arrays and highlight these arrays may be favourable by providing a larger window to study migration kinetics and the added advantage in the 96-well format; thus, adding greater pharmacological power to experimental design.

An ideal addendum to this technology is live-cell imaging in concert with the ECIS measurement to see exactly what is occurring at the cellular level and when. The imaging data we have shown from the ablation experiments is immuno-fluorescent staining of cells post-fixation, this is quite different to the power of live-cell imaging. However, we are not aware of routine live-cell imaging systems that are readily compatible with ECIS technology. There are technical hurdles to overcome including the positioning of the objectives in the up-right position above the wells. This is limited by the well height on the array, working distance of the objectives, and stage depth to contain the ECIS array holder. The ability to conduct live-cell imaging in concert with real-time ECIS measurement would be highly valuable for downstream applications such as cytotoxicity assays or NK-mediated killing of the glioblastoma cells.

We can conclude from our observations, that these ECIS arrays are suitable for ablation of adherent glioblastoma cells and measurement of migration on to the electrodes. ECIS clearly provides a valuable time frame for migration to occur, where the electrode resistance appears to correlate well with migration.

## 5. Conclusions

ECIS technology was not capable of measuring the weak level of adhesion from the glioblastoma stem cell cultures but was very capable of detecting and monitoring the strong level of adhesion from the serum-derived glioblastoma cells. This opens considerable opportunities for using ECIS technology in future studies investigating (i) mechanism of killing glioblastoma cells, and (ii) migratory kinetics.

## Figures and Tables

**Table 1:** NanoString gene expression of adhesion molecules. Table shows the respective gene of interest, the NCBI accession number and the target gene sequence detected. The top 4 genes in the table are house-keeping genes. The remainder are key integrin family members and cadherins.

Gene	Accession	Target Sequence
	Housekeepers	
***MRPS5***	NM_031902.3	ATCCCTACGCCAGCTTGAGCCGTGCACTGCAGACACAATGCTGTATTTCTTCTCCCAGTCACCTGATGAGCCAGCAGTATAGACCATATAGTTTCTTCAC
***PCNA***	NM_002592.2	GGTGTTGGAGGCACTCAAGGACCTCATCAACGAGGCCTGCTGGGATATTAGCTCCAGCGGTGTAAACCTGCAGAGCATGGACTCGTCCCACGTCTCTTTG
***PPIA***	NM_021130.3	TCTATGGGGAGAAATTTGAAGATGAGAACTTCATCCTAAAGCATACGGGTCCTGGCATCTTGTCCATGGCAAATGCTGGACCCAACACAAATGGTTCCCA
***TBP***	NM_001172085.1	ACAGTGAATCTTGGTTGTAAACTTGACCTAAAGACCATTGCACTTCGTGCCCGAAACGCCGAATATAATCCCAAGCGGTTTGCTGCGGTAATCATGAGGA
***CDH1***	NM_001317184.1	CCTTCCTCCCAATACATCTCCCTTCACAGCAGAACTAACACACGGGGCGAGTGCCAACTGGACCATTCAGTACAACGACCCAACCCAAGAATCTATCATT
***CDH11***	NM_001797.2	CAGGAAGCCAAAGTCCCAGTGGCCATTAGGGTCCTTGATGTCAACGATAATGCTCCCAAGTTTGCTGCCCCTTATGAAGGTTTCATCTGTGAGAGTGATC
***CDH12***	NM_004061.3	CGCCTAATCTTCACCCGCTGCTAGGCTCGTTTAATGAGTCTTCTGAGAGCTAAGGAGTCCTCGGATTCATTCAAAGCATTCTACAATGAACGCTAGGGGG
***CDH13***	NM_001220488.1	ATCTGCCATGCAAAACGAGGGAGCGTTAGGAAGGAATCCGTCTTGTAAAGCCATTGGTCCTGGTCATCAGCCTCTACCCAATGCTTTCGTGATGCTGCTG
***CDH19***	NM_021153.2	TCCAGAAGGAACATTAGTTATCCAGGTGACAGCAAGTGATGCTGACGATCCCTCAAGTGGTAATAATGCTCGTCTCCTCTACAGCTTACTTCAAGGCCAG
***CDH2***	NM_001792.3	GGTCATCCCTCCAATCAACTTGCCAGAAAACTCCAGGGGACCTTTTCCTCAAGAGCTTGTCAGGATCAGGTCTGATAGAGATAAAAACCTTTCACTGCGG
***CDH24***	NM_022478.3	TCCTCTCCTTCCTCCGTGGCGTTTTGTCTCTGCAGTTCTGAAGCTCACACATAGTCTCCCTGCGTCTTCCTTGCCCATACACATGCTCTGTGTCTGTCTC
***CDH3***	NM_001317195.1	AGCTCTGTTTAGCACTGATAATGATGACTTCACTGTGCGGAATGGCGAGACAGTCCAGGAAAGAAGGTCACTGAAGGAAAGGAATCCATTGAAGATCTTC
***CDH4***	NM_001794.2	AGAGAAAGTTCAGCAGTACACAGTCATCGTTCAGGCCACAGATATGGAAGGAAATCTCAACTATGGCCTCTCAAACACAGCCACAGCCATCATCACGGTG
***CDH5***	NM_001795.3	TCTCCCCTTCTCTGCCTCACCTGGTCGCCAATCCATGCTCTCTTTCTTTTCTCTGTCTACTCCTTATCCCTTGGTTTAGAGGAACCCAAGATGTGGCCTT
***CDH7***	NM_004361.2	GTTACACGCTACGGATAGAAGCTGCAAATAAAGATGCCGACCCTCGCTTTCTGAGCTTGGGTCCGTTCAGTGACACGACAACTGTGAAGATAATTGTGGA
***ITGA1***	NM_181501.1	AAGTGGCAAGACTATAAGGAAAGAGTATGCACAACGTATTCCATCAGGTGGGGATGGTAAGACACTGAAATTTTTTGGCCAGTCTATCCACGGAGAAATG
***ITGA11***	NM_012211.3	CCTGAAAAAGTTTTACATTGGCCCAGGGCAGATCCAGGTTGGAGTTGTGCAGTATGGCGAAGATGTGGTGCATGAGTTTCACCTCAACGACTACAGGTCT
***ITGA2***	NM_002203.2	CAACGGGTGTGTGTTCTGACATCAGTCCTGATTTTCAGCTCTCAGCCAGCTTCTCACCTGCAACTCAGCCCTGCCCTTCCCTCATAGATGTTGTGGTTGT
***ITGA2B***	NM_000419.3	AGTTACCGCCCAGGCATCCTTTTGTGGCACGTGTCCTCCCAGAGCCTCTCCTTTGACTCCAGCAACCCAGAGTACTTCGACGGCTACTGGGGGTACTCGG
***ITGA3***	NM_005501.2	CATGATTCAGCGCAAGGAGTGGGACTTATCTGAGTATAGTTACAAGGACCCAGAGGACCAAGGAAACCTCTATATTGGGTACACGATGCAGGTAGGCAGC
***ITGA4***	NM_000885.4	GCCCACTGCCAACTGGCTCGCCAACGCTTCAGTGATCAATCCCGGGGCGATTTACAGATGCAGGATCGGAAAGAATCCCGGCCAGACGTGCGAACAGCTC
***ITGA5***	NM_002205.2	AGAAGACTTTGTTGCTGGTGTGCCCAAAGGGAACCTCACTTACGGCTATGTCACCATCCTTAATGGCTCAGACATTCGATCCCTCTACAACTTCTCAGGG
***ITGA6***	NM_000210.1	CTCATGCGAGCCTTCATTGATGTGACTGCTGCTGCCGAAAATATCAGGCTGCCAAATGCAGGCACTCAGGTTCGAGTGACTGTGTTTCCCTCAAAGACTG
***ITGA9***	NM_002207.2	CATGTCTCCAACCTCCTTTGTATATGGCGAGTCCGTGGACGCAGCCAACTTCATTCAGCTGGATGACCTGGAGTGTCACTTTCAGCCCATCAATATCACC
***ITGAD***	NM_005353.2	ATTGACGGCTCTGGAAGCATTGACCAAAATGACTTTAACCAGATGAAGGGCTTTGTCCAAGCTGTCATGGGCCAGTTTGAGGGCACTGACACCCTGTTTG
***ITGAE***	NM_002208.4	CTGAATGCAGAGAACCACAGAACTAAGATCACTGTCGTCTTCCTGAAAGATGAGAAGTACCATTCTTTGCCTATCATCATTAAAGGCAGCGTTGGTGGAC
***ITGAL***	NM_001114380.1	GCAGGATGACACATTTATTGGGAATGAACCATTGACACCAGAAGTGAGAGCAGGCTATTTGGGTTACACCGTGACCTGGCTGCCCTCCCGGCAAAAGACT
***ITGAM***	NM_000632.3	GCCCTCCGAGGGTGTCCTCAAGAGGATAGTGACATTGCCTTCTTGATTGATGGCTCTGGTAGCATCATCCCACATGACTTTCGGCGGATGAAGGAGTTTG
***ITGAV***	NM_002210.2	TTTCTTCCGATTCCAAACTGGGAGCACAAGGAGAACCCTGAGACTGAAGAAGATGTTGGGCCAGTTGTTCAGCACATCTATGAGCTGAGAAACAATGGTC
***ITGAX***	NM_000887.3	CCCCTCAGCCTGTTGGCTTCTGTTCACCAGCTGCAAGGGTTTACATACACGGCCACCGCCATCCAAAATGTCGTGCACCGATTGTTCCATGCCTCATATG
***ITGB1***	NM_033666.2	TTTTAACATTACCAAGGTAGAAAGTCGGGACAAATTACCCCAGCCGGTCCAACCTGATCCTGTGTCCCATTGTAAGGAGAAGGATGTTGACGACTGTTGG
***ITGB2***	NM_000211.2	CATCGACCTGTACTATCTGATGGACCTCTCCTACTCCATGCTTGATGACCTCAGGAATGTCAAGAAGCTAGGTGGCGACCTGCTCCGGGCCCTCAACGAG
***ITGB3***	NM_000212.2	GAATAAGCCTTGGAATTAGATATGGGGCAATGACTGAGCCCTGTCTCACCCATGGATTACTCCTTACTGTAGGGAATGGCAGTATGGTAGAGGGATAAAT
***ITGB4***	NM_001005731.1	GGCCCATGTCCATCCCCATCATCCCTGACATCCCTATCGTGGACGCCCAGAGCGGGGAGGACTACGACAGCTTCCTTATGTACAGCGATGACGTTCTACG
***ITGB5***	NM_002213.3	TAATCTCTTCTTTACTGCTACCTGCCAAGATGGGGTATCCTATCCTGGTCAGAGGAAGTGTGAGGGTCTGAAGATTGGGGACACGGCATCTTTTGAAGTA
***ITGB6***	NM_000888.3	AACATTCTCCAGCTGATCATCTCAGCTTATGAAGAACTGCGGTCTGAGGTGGAACTGGAAGTATTAGGAGACACTGAAGGACTCAACTTGTCATTTACAG
***ITGB7***	NM_000889.1	CAACGTGGTACAGCTCATCATGGATGCTTATAATAGCCTGTCTTCCACCGTGACCCTTGAACACTCTTCACTCCCTCCTGGGGTCCACATTTCTTACGAA
***ITGB8***	NM_002214.2	GGAAAACTGGAATTGTATGCAATGCCTTCACCCTCACAATTTGTCTCAGGCTATACTTGATCAGTGCAAAACCTCATGTGCTCTCATGGAACAACAGCAT

## Data Availability

Original data from this study is held by the lead author E Scott Graham and can be obtained by request at s.graham@auckland.ac.nz.

## Supplementary Materials

The following are available online at https://www.mdpi.com/article/10.3390/bios11120498/s1, Figure S1 Serum-derived and gCSC cell resistance on 96W1E+ ECIS arrays. Phase contrast images of NZB11, NZB12, NZB19, and NZB13 serum-derived and gCSC cells after 60 h of growth on 96W1E+ ECIS arrays. Images acquired at 20× magnification. Data are representative of three independent replicates.
