# Supplementary material for: Can ECIS Biosensor Technology Be Used to Measure the Cellular Responses of Glioblastoma Stem Cells?
Source: Biosensors (Basel). 2021 Dec 6;11(12):498. doi: 10.3390/bios11120498 (PMC8699647; doi:10.3390/bios11120498)
Supplement: Supplementary file 1 [file biosensors-11-00498-s001.zip › biosensors-1447619-supplementary.pdf]

biosensors

Article

Can ECIS Biosensor Technology Be Used to Measure the Cellular Responses of Glioblastoma Stem cells?

Laverne Diana Robilliard [1,2,†], Jane Yu [1,2,†], Sung-Min Jun [1,2], Akshata Anchan [1,2], Graeme Finlay [1,3], Catherine E. Angel [4] and Euan Scott Graham [1,2,*]

1 Department of Molecular Medicine and Pathology, School of Medical Sciences, Faculty of Medical and Health Sciences, University of Auckland, Auckland 1025, North Island, New Zealand; l.robilliard@auckland.ac.nz (L.D.R.); jane.yu@auckland.ac.nz (J.Y.); sjun881@aucklanduni.ac.nz (S.-M.J.); a.anchan@auckland.ac.nz (A.A.); g.finlay@auckland.ac.nz (G.F.)
2 Centre for Brain Research, University of Auckland, Auckland 1025, North Island, New Zealand
3 Auckland Cancer Society Research Centre, Faculty of Medical and Health Sciences, University of Auckland,Auckland 1025, North Island, New Zealand
4 School of Biological Sciences, Faculty of Science, University of Auckland, Auckland 1025, North Island, New Zealand; c.angel@auckland.ac.nz
* Correspondence: s.graham@auckland.ac.nz
† These authors contributed equally to this work.

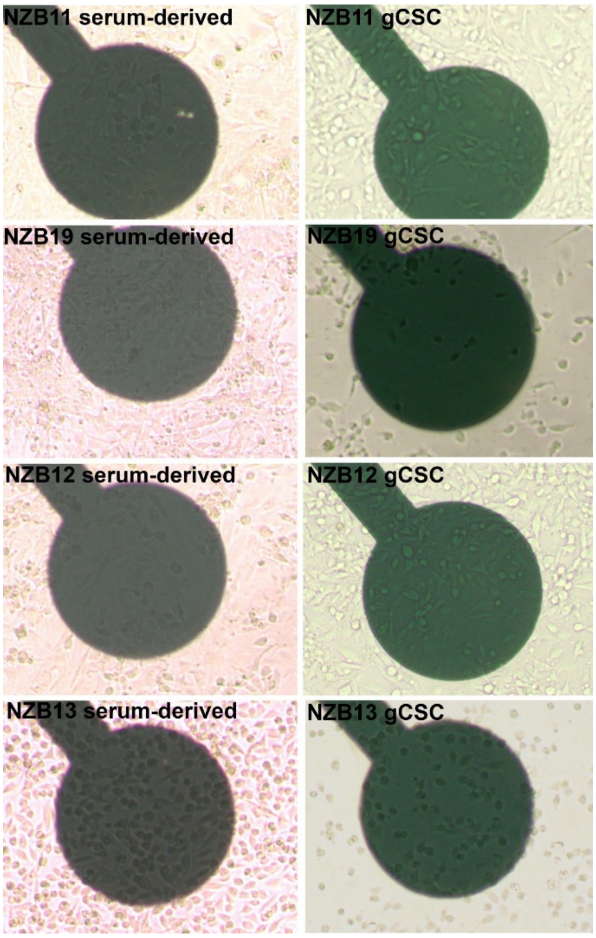

Figure S1 Serum-derived and gCSC cell resistance on 96W1E+ ECIS arrays. Phase contrast images of NZB11, NZB12, NZB19, and NZB13 serum-derived and gCSC cells after 60 h of growth on 96W1E+ ECIS arrays. Images acquired at 20× magnification. Data are representative of three independent replicates.
